# Bridging Microbial Biocontrol and Phytochemical Biopesticides: Synergistic Approaches for Sustainable Crop Protection

**DOI:** 10.3390/plants14223453

**Published:** 2025-11-12

**Authors:** Younes Rezaee Danesh, Jose Miguel Mulet, Rosa Porcel

**Affiliations:** 1 Department of Plant Protection, Faculty of Agriculture, Van Yuzuncu Yil University, Van 65090, Türkiye; 2 Instituto de Biología Molecular y Celular de Plantas, Universitat Politècnica de València-Consejo Superior de Investigaciones Científicas, 46022 Valencia, Spain; roporrol@upv.es

**Keywords:** microbial biocontrol, phytochemical biopesticides, sustainable agriculture, crop protection, synergistic approaches

## Abstract

The increasing prevalence of pests and diseases in agriculture necessitates innovative strategies for crop protection that mitigate environmental impacts. This review paper investigates the synergistic potential of combining microbial biocontrol agents and phytochemical biopesticides as sustainable alternatives to chemical pesticides. Through a comprehensive review of recent literature, we analyze the mechanisms by which beneficial microbes (e.g., *Trichoderma*, *Bacillus*, and *Pseudomonas*) enhance plant resilience and suppress pathogens, and how plant-derived phytochemicals such as essential oils, alkaloids, and flavonoids contribute to pest deterrence and disease resistance. The integration of these bio-based resources forms an actionable framework for sustainable crop protection—enabling reduced chemical dependence, improved soil health, and enhanced biodiversity. Examples of synergistic success, such as the combined use of Bacillus thuringiensis with neem extract and Trichoderma with lemongrass oil, illustrate their field potential. Future research should prioritize the formulation of stable microbial–phytochemical consortia, field validation of synergistic efficacy, and optimization of delivery systems to support commercial-scale adoption. Ultimately, this study promotes a paradigm shift toward eco-efficient pest management, bridging fundamental research and applied innovation for resilient agroecosystems.

## 1. Introduction to Sustainable Crop Protection

Sustainable crop protection encompasses the systematic management of biological, chemical, and physical factors that collectively influence crop development, yield, and quality, while striving to minimize adverse environmental and societal impacts. According to Montesinos [1], sustainable agriculture can be characterized as a system that effectively utilizes biocontrol agents, including natural microbial antagonists for disease and pest suppression, as well as biopesticides derived from natural sources rather than synthetic chemicals [2]. Consequently, modern pest management extends beyond conventional practices, emphasizing the central role of microorganisms in enhancing the overall health, resilience, and productivity of crop plants [3]. In pest management, a synergistic combination of microbial biocontrol agents and phytochemical biopesticides provides several advantages for sustainable crop protection. Such integrated strategies can enhance pathogen suppression, reduce the emergence of resistance, and maintain ecological balance compared to single-agent applications. For instance, the combined use of *Bacillus thuringiensis* with neem (*Azadirachta indica*) extracts has shown improved control of lepidopteran pests while reducing the required microbial dose [4,5]. Similarly, formulations combining *Trichoderma harzianum* with essential oils of *Cymbopogon citratus* (lemongrass) or *Rosmarinus officinalis* (rosemary) significantly improved antifungal efficacy against *Botrytis cinerea* and *Rhizoctonia solani* [6,7]. These examples demonstrate that synergistic approaches not only increase biocontrol performance but also promote long-term sustainability through reduced chemical input and improved environmental safety [8,9]. This sustainable approach embraces environmentally friendly methodologies, integrating pest and nutrient management techniques with comprehensive pollution control measures and prudent conservation of critical agricultural resources [10]. By emphasizing these eco-conscious strategies, we can cultivate a more harmonious agricultural ecosystem, ensuring that farming practices not only enhance productivity but also safeguard the health of our planet and the well-being of future generations. In recent years, increasing attention has been devoted to the exploration of alternative bio-based resources for sustainable plant protection. Advanced biotechnological approaches have facilitated major progress in microbial biocontrol, employing beneficial microorganisms to suppress plant pests and pathogens through diverse mechanisms. Concurrently, plant-derived phytochemicals have demonstrated potent antimicrobial, insecticidal, and nematicidal activities that can effectively combat destructive plant diseases responsible for substantial yield losses. The integration of microbial and phytochemical biocontrol strategies offers a more efficient, sustainable, and holistic pathway toward crop protection. Such synergistic combinations can enhance efficacy, broaden the pest-control spectrum, and minimize dependence on synthetic agrochemicals—thereby aligning with global efforts to achieve environmentally resilient and economically viable agriculture. This review provides an integrative perspective that bridges microbial biocontrol and phytochemical biopesticide research—two fields often treated separately. By synthesizing current mechanistic insights, field data, and regulatory considerations, it outlines a unified conceptual and practical framework for developing synergistic biocontrol systems. The review highlights recent evidence showing that microbial–phytochemical combinations can outperform single-agent applications in pathogen suppression, while also identifying existing limitations in formulation stability, regulatory alignment, and farmer adoption. These insights offer a foundation for future innovations in sustainable pest management and precision agriculture.

## 2. Overview of Microbial Biocontrol Agents

**Figure 1:**
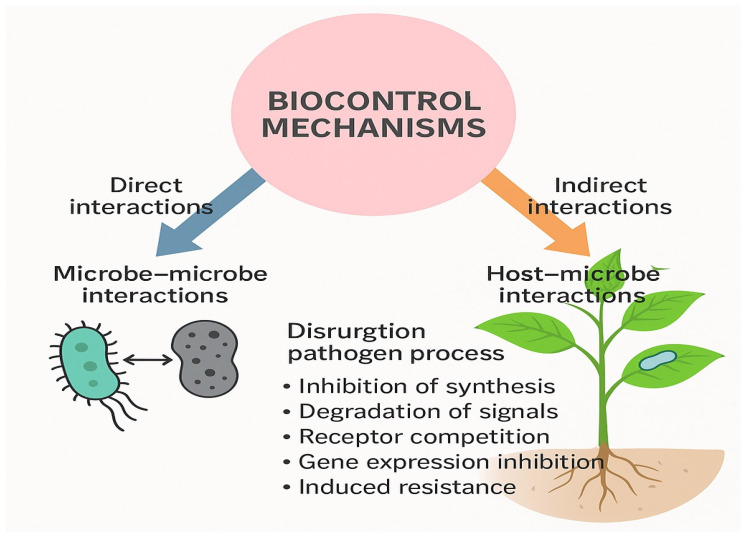
Biocontrol mechanisms in plant disease management, categorized into direct interactions (microbe–microbe interactions) and indirect interactions (host–microbe interactions). These mechanisms disrupt pathogen processes through inhibition of signaling molecule synthesis, degradation of signals, receptor competition, gene expression inhibition, and induction of plant systemic resistance.

Microorganisms play a crucial role in the biological control of plant diseases, although some can also act as pathogens. Microbial biocontrol agents (BCAs) suppress a wide range of plant pathogens and enhance plant resistance against both biotic and abiotic stresses. Major groups of BCAs include bacteria such as *Pseudomonas* spp. and *Bacillus* spp., fungi such as *Trichoderma* spp., and viruses that specifically target harmful phytopathogens or insect pests. These microorganisms employ multiple mechanisms of action, including antibiosis (production of inhibitory substances), parasitism (direct exploitation of other organisms), predation (consumption of target organisms), and competition for nutrients and ecological niches. Additionally, many BCAs induce systemic plant defense responses, thereby improving plant resilience to disease [11] (Figure 1). Microbial BCAs offer several advantages: they exhibit broad-spectrum activity, are compatible with integrated pest management (IPM) programs, display high target specificity, are safe for non-target organisms, and comply with organic farming standards, making them attractive tools for sustainable agriculture [12]. However, their field efficacy may vary under different environmental conditions due to complex interactions with indigenous soil and plant microbiota [13]. Developing reliable, long-term application strategies remains an ongoing challenge that continues to attract intensive research efforts [8,14].

### 2.1. Types of Microbial Biocontrol Agents

Microbial biocontrol agents (MBAs) represent an alternative to conventional crop-protection strategies that offer improved environmental sustainability [15]. In our view, microbial biocontrol agents represent a superior and more sustainable approach to plant protection compared with conventional chemical methods. They not only suppress pathogens effectively through diverse mechanisms—such as antibiosis, parasitism, and induction of systemic resistance—but also improve soil microbiome balance and plant vigor while reducing chemical residues and environmental toxicity. However, we acknowledge that chemical pesticides may still be necessary under certain outbreak or emergency conditions due to their rapid curative action. Hence, the integration of microbial biocontrol agents within an integrated pest-management (IPM) framework provides the most effective and ecologically balanced strategy, combining immediate efficacy with long-term environmental safety [9,11,12,14]. Several types of microbial agents are widely employed as biocontrol tools, among which *Bacillus*, *Pseudomonas*, and *Trichoderma* species are the most relevant—together accounting for nearly 80% of the global biopesticide market—alongside mycorrhizal fungi and endophytic microorganisms. *Bacillus* spp. include both saprophytic and facultative endophytic bacteria that promote plant growth by fixing atmospheric nitrogen, solubilizing phosphates, and producing extracellular hydrolases that degrade pathogen cell walls. They also synthesize a wide range of secondary metabolites with antibacterial, antifungal, antiviral, and nematicidal properties, notably cyclic lipopeptides and polyketides, which play key roles in crop protection [14,16]. Commonly used biocontrol species include *Bacillus subtilis*, *Bacillus amyloliquefaciens*, *Bacillus velezensis*, and *Bacillus thuringiensis*. *Pseudomonas* species have long been recognized as efficient biocontrol agents against diverse crop diseases. These bacteria are predominantly saprophytic but can also colonize both the rhizosphere and internal plant tissues. Their main mechanisms of action include the production of pathogenesis-related (PR) proteins that stimulate plant immune responses, competition for nutrients and ecological niches, and the synthesis of diverse bioactive secondary metabolites. These include cyclic lipopeptides, siderophores, and antibiotics such as 2,4-diacetylphloroglucinol (DAPG), along with phytohormones, hydrolytic enzymes, and insecticidal toxins. *Trichoderma* species are among the most important genera of filamentous fungi utilized in commercial biocontrol products. Their mechanisms involve nutrient solubilization, production of secondary metabolites similar to those produced by *Bacillus* and *Pseudomonas*, and induction of systemic resistance in host plants [17]. This genus encompasses both saprophytic and facultative endophytic fungi, with species such as *Trichoderma virens*, *Trichoderma harzianum*, and *Trichoderma atroviride* being widely used across numerous crops. Arbuscular mycorrhizal fungi (AMF) are obligate symbionts that form mutualistic associations with the roots of more than 80% of terrestrial plants. In addition to improving nutrient acquisition, AMF elicit plant immune responses through signaling molecules such as jasmonic acid, salicylic acid, and ethylene [18,19,20]. Mycorrhizal fungi are classified as arbuscular (AMF) or ectomycorrhizal (ECM), with AMF being more commonly used in biocontrol. Endophytic microorganisms, which inhabit plant tissues without causing damage, contribute to plant resistance against both biotic and abiotic stresses. Several studies have demonstrated the successful use of AMF and other endophytes in reducing fungal diseases in crops such as strawberry [21]. These microorganisms enhance plant performance by supplying nutrients, synthesizing phytohormones and bioactive compounds, inducing systemic resistance, and competing with pathogens for space and resources. The successful development and performance of microbial biocontrol agents depend on several interrelated factors. Intrinsic microbial traits such as growth rate, sporulation capacity, genetic stability, and ability to colonize plant tissues or the rhizosphere directly influence field efficacy. Environmental conditions—including soil type, moisture, temperature, and pH—affect microbial survival, activity, and metabolite production [13,14]. The presence of native microbiota and competition for space or nutrients can also modulate biocontrol effectiveness. Furthermore, technological factors such as formulation design, carrier materials, and storage stability play crucial roles in maintaining microbial viability and shelf life [22,23]. Finally, regulatory and agronomic practices, such as pesticide compatibility and application timing, determine the overall adoption and integration of biocontrol strategies in crop production systems [1,9]. Understanding these parameters is essential to optimize microbial selection, formulation, and deployment under field conditions and to ensure reproducible biocontrol outcomes.

### 2.2. Mechanisms of Action

The utilization of microbial biocontrol agents (BCAs) plays a crucial role in suppressing plant pathogens and enhancing overall plant resilience. A growing body of literature has extensively investigated the molecular mechanisms of pathogenesis that BCAs target or disrupt. Many phytopathogens depend on a complex communication system known as quorum sensing (QS), which enables them to coordinate the production of virulence factors. This process involves the accumulation of diffusible signaling molecules that, once reaching a threshold concentration, bind to specific receptors and trigger the expression of genes responsible for pathogenicity [24]. Among the various mechanisms of microbial biocontrol, induction of systemic resistance (ISR) represents one of the most critical and biologically sophisticated processes. ISR is a plant-mediated defense mechanism activated by certain beneficial microbes such as *Pseudomonas fluorescens*, *Bacillus subtilis*, and *Trichoderma harzianum*. These microorganisms trigger host immune pathways through signaling molecules like jasmonic acid, ethylene, and salicylic acid, thereby “priming” plants for faster and stronger responses upon pathogen attack. Unlike direct antagonism, ISR does not rely on pathogen presence but rather enhances the plant’s intrinsic immunity, leading to reduced disease incidence and improved tolerance against multiple pathogens simultaneously [18,19]. Other mechanisms also contribute significantly to biocontrol efficacy. Antibiosis involves the production of antibiotics, lipopeptides, or volatile compounds (e.g., 2,4-diacetylphloroglucinol, iturin, and surfactin) that inhibit pathogen growth. Parasitism and mycoparasitism, characteristic of fungi like *Trichoderma* spp., entail direct attack and enzymatic degradation of pathogen cell walls through chitinases and glucanases. Competition for nutrients and ecological niches prevents pathogens from establishing dominance in the rhizosphere. Finally, quorum quenching—the disruption of pathogen communication signals—has emerged as a novel strategy where beneficial bacteria degrade or neutralize quorum-sensing molecules, thereby suppressing virulence gene expression [24,25]. Collectively, these mechanisms operate singly or synergistically, ensuring the robustness and versatility of microbial biocontrol agents in sustainable crop protection. In response to pathogen quorum sensing, microorganisms have evolved diverse strategies to disrupt or inhibit this communication system, thereby attenuating pathogen virulence. These mechanisms include inhibition of signal-molecule synthesis, enzymatic degradation or deactivation of signaling compounds, antibody binding or competitive receptor occupation, and suppression of quorum-regulated gene expression that would otherwise promote pathogenicity [25]. The use of microbial consortia has emerged as a promising approach, harnessing complex interspecies interactions and trophic networks to enhance the efficacy of biocontrol systems [13]. However, challenges remain, including potential incompatibility among microbial partners, unpredictable ecological interactions, and antagonistic effects of antimicrobial compounds produced by certain consortium members on others. The phyllosphere—comprising diverse bacterial, yeast, and fungal communities on aerial plant surfaces—represents a highly dynamic habitat. These microorganisms exhibit remarkable adaptability to environmental fluctuations and frequently display antagonistic activity against opportunistic pathogens. Biocontrol processes can be broadly divided into direct microbe–microbe interactions and indirect host–microbe interactions involving the plant itself (Figure 1). A comprehensive understanding of the genetic and molecular bases of these mechanisms, supported by well-designed laboratory and field experiments, is crucial for the rational design and formulation of effective microbial consortia. Such approaches aim to achieve reliable disease suppression without compromising plant growth or productivity, thereby reconciling the dual challenge of maintaining high agricultural output while minimizing environmental impact in the face of global population growth. In addition, many beneficial microorganisms can induce systemic resistance (ISR) in plants, enhancing immunity through elicitor production or endophytic colonization. They trigger hormonal crosstalk and signal-transduction cascades that activate key defense-related genes, effectively priming plants against both pathogens and pests [14,26].

### 2.3. Advantages and Limitations

Microbial biocontrol agents (BCAs)—including fungi, bacteria, and viruses—provide effective and environmentally friendly alternatives for controlling a wide range of phytopathogens, nematodes, and insect pests. They exhibit high target specificity and low toxicity toward non-target organisms, making them valuable tools for sustainable crop protection. Their principal modes of action include antibiosis, competition, parasitism, and induced systemic resistance (ISR) [14]. Despite these advantages, microbial agents present several limitations. Their narrow host specificity restricts application to specific pests and diseases, necessitating accurate diagnosis. In addition, complex and costly processes involved in mass culturing, storage, and formulation hinder large-scale commercialization [22]. Furthermore, their field efficacy is often influenced by environmental factors such as temperature, humidity, sunlight, and soil pH, which can reduce consistency and reliability under diverse agricultural conditions [1,27]. The advantages and limitations of microbial biocontrol agents can be systematically compared to guide their integration into sustainable pest-management programs. Table 1 summarizes the main benefits and constraints documented across recent studies.

**Table 1:** Main advantages and limitations of microbial biocontrol agents (MBCAs).

Aspect	Advantages	Limitations/Challenges	Representative References
Efficacy & Specificity	Target-specific pathogen control; minimal impact on beneficial organisms	Narrow host range; reduced effectiveness under extreme field conditions	[1,14,22]
Environmental Impact	Biodegradable, non-toxic residues, low ecological risk	Short persistence in soil; variable survival after application	[9,11,27]
Mechanistic Diversity	Multiple actions—antibiosis, competition, induced systemic resistance, mycoparasitism	Inter-strain incompatibility; unpredictable interaction effects	[18,19,24]
Economic Feasibility	Reduced long-term chemical costs; suited for organic markets	High production/formulation costs; limited shelf life	[22,28]
Adoption & Regulation	Growing market and policy support for sustainable tools	Complex registration procedures; inconsistent international frameworks	[9,29]

Recent literature further emphasizes that optimization of formulation technologies, encapsulation materials, and carrier systems can mitigate some of these constraints and improve field reliability [23,24,28]. Continued research integrating microbial and phytochemical agents offers opportunities to overcome stability and efficacy limitations while maintaining environmental safety. Phytochemical biopesticides, derived primarily from crude plant extracts and essential oils, exhibit broad-spectrum activity against diverse pathogens, including bacteria, fungi, viruses, insects, and nematodes. They function as biodegradable, eco-friendly alternatives to conventional synthetic agrochemicals, offering both safety and efficacy in sustainable crop protection [30].

## 3. Phytochemical Biopesticides: An Overview

Plants are an abundant source of bioactive phytochemicals that display a wide range of biological activities. Many modern agrochemicals have been developed from these natural compounds, which play essential roles in plant defense and ecological interactions. Among them, secondary metabolites such as alkaloids, flavonoids, phenolics, saponins, and essential oils function as natural antifeedants and repellents, protecting plants from herbivores and insect pests [31]. The development of phytochemical biopesticides typically involves several stages: collection of raw plant material, drying to preserve chemical integrity, and extraction of active compounds using suitable solvents such as methanol or acetone to isolate the desired bioactive constituents [32]. These plant-derived products are increasingly viewed as safer and more sustainable alternatives to conventional synthetic pesticides. Currently, approximately 95% of global pesticides are chemically synthesized, posing substantial risks to human health, animals, and the environment [33]. In contrast, phytochemical biopesticides offer multiple advantages, including biodegradability, low production costs, and high availability of raw materials. They are generally less toxic to mammals and compatible with integrated pest management (IPM) systems, thereby supporting sustainable agricultural practices [8,9]. Through the implementation of such eco-friendly solutions, agriculture can advance toward a more resilient and environmentally responsible future. Common plant-based biopesticides are derived from several key botanical sources and demonstrate a wide spectrum of bioactivities. Neem (*Azadirachta indica*) extracts containing azadirachtin act as potent antifeedants, insect growth regulators, and oviposition deterrents. Pyrethrum obtained from *Chrysanthemum cinerariifolium* flowers targets insect nervous systems by prolonging sodium channel activation, leading to paralysis. Rotenone, isolated from *Derris* and *Lonchocarpus* species, interferes with mitochondrial electron transport, exhibiting strong insecticidal properties. Essential oils from plants such as *Cymbopogon citratus* (lemongrass), *Thymus vulgaris* (thyme), and *Eucalyptus globulus* demonstrate antifungal and antibacterial activities through membrane disruption and inhibition of spore germination. Alkaloids (e.g., nicotine, caffeine), phenolics, and saponins also contribute to pest suppression by acting as deterrents, enzyme inhibitors, or signaling disruptors. Together, these natural compounds exemplify diverse modes of action—including neurotoxicity, growth inhibition, repellency, and oxidative stress induction—that make phytochemical biopesticides promising tools in integrated pest management programs [9,31,32,33].

### 3.1. Sources of Phytochemicals

**Figure 2:**
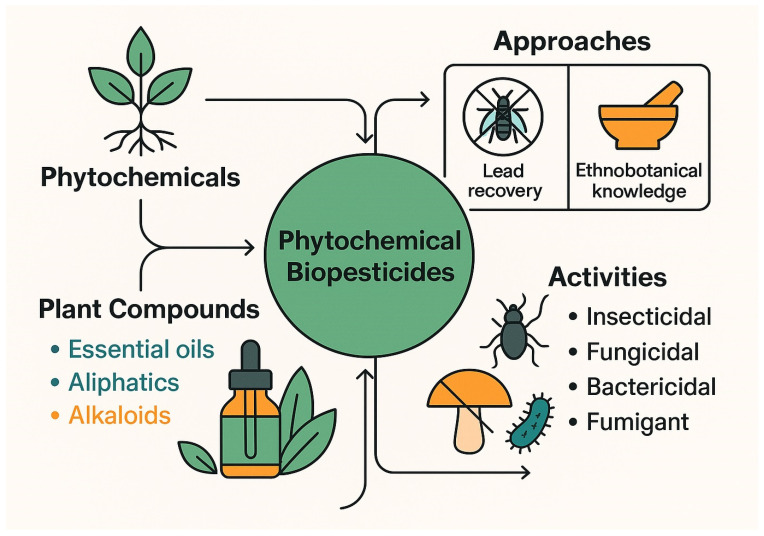
Conceptual framework of phytochemical biopesticides showing their plant-derived origin, major compound classes (essential oils, aliphatics, alkaloids), two main bioprospecting approaches (lead recovery and ethnobotanical knowledge), and their broad-spectrum biological activities (insecticidal, fungicidal, bactericidal, and fumigant).

Phytochemicals are natural plant-derived compounds that function as primary or secondary metabolites, providing protection against diverse environmental and biological stressors. As a key component of integrated pest management (IPM) programs, phytochemical biopesticides are typically safer and more selective toward target pests than conventional synthetic pesticides [14] (Figure 2). Bioprospecting for novel phytochemical agents generally follows two approaches. The first involves the isolation of phytochemical leads responsible for specific biological activities such as pest repellency—for example, azadirachtin, the active compound in neem-based repellents. The second relies on ethnobotanical knowledge, utilizing traditional plant preparations with proven efficacy against agricultural pests [15]. Phytochemical biopesticides can be obtained from various plant matrices, with the most potent compounds often derived from medicinal herbs. Waste materials rich in essential oils are also valuable sources of bioactive constituents, which may be combined in specific proportions to enhance efficacy. The main classes of bioactive phytochemicals include aliphatic and aromatic compounds, essential oils, and alkaloids, which disrupt enzymatic activity and the nervous systems of pests. Essential oils, present in nearly all plant organs, play an important role in natural defense against herbivores and pathogens. Their biological activity is largely attributed to interactions with the lipid membranes of target organisms, leading to cell lysis and death. Phytochemical extracts and purified fractions exhibit strong insecticidal, fungicidal, bactericidal, and fumigant activities, while maintaining rapid biodegradability. Most of these compounds show broad-spectrum efficacy against agricultural pests and decompose readily into environmentally benign products [34].

### 3.2. Extraction and Formulation Techniques

Solvent extraction remains one of the most widely used methods for recovering essential oils from aromatic plants, complemented by modern alternatives such as supercritical carbon dioxide extraction. The choice of recovery system largely depends on the physical and chemical characteristics of the raw material. While crude vegetable oils are typically processed using solvent extraction, the recovery of used cooking oils and industrial residues often relies on adsorption and membrane-based techniques. Over the years, several technological approaches have been developed for essential-oil extraction, including solvent extraction, steam distillation, enfleurage, and maceration. Each method offers distinct advantages in yield, purity, and suitability for different plant matrices, enabling more efficient recovery of valuable phytochemical constituents. Various extraction methods have been developed and optimized for different medicinal and aromatic plants. Steam distillation remains the most widely applied technique for plants such as *Cymbopogon citratus* (lemongrass), *Eucalyptus globulus* (eucalyptus), and *Rosmarinus officinalis* (rosemary), typically yielding 0.3–2.0% (*v*/*w*) of essential oils depending on the plant part and harvest stage. Solvent extraction is frequently used for *Azadirachta indica* (neem) seeds, producing 20–25% (*w*/*w*) crude oil rich in azadirachtin, though solvent residues and high energy requirements limit its sustainability. Hydrodistillation provides moderate yields (0.5–1.5%) from species such as *Thymus vulgaris* (thyme) and *Mentha piperita* (peppermint) but requires substantial water input. More recently, supercritical CO_2_ extraction has been adopted for high-value plants including *Lavandula angustifolia* (lavender) and *Matricaria chamomilla* (chamomile), achieving up to 3% yields with minimal solvent residues and superior purity. This method is considered more sustainable due to low solvent toxicity and reusability, despite higher initial equipment costs. Overall, the choice of extraction technique depends on plant species, target compounds, and desired purity. Sustainable extraction approaches prioritize reduced energy and solvent consumption, recyclability, and scalability, which are essential for the eco-friendly production of phytochemical biopesticides [30,31,33]. Enfleurage is rarely applied on an industrial scale, and the volatile solvents commonly used in conventional solvent extraction are often hazardous and environmentally harmful. Although steam distillation remains the most widely adopted method, it presents several drawbacks, including long processing times and high energy consumption [1]. To overcome these limitations, solvent-free microwave extraction has emerged as a novel and eco-friendly alternative, offering higher product quality, shorter extraction times, and greater energy efficiency [35,36]. Parallel advancements in biocontrol formulation technologies have introduced diverse options such as liquid formulations, encapsulation, spray drying, and freeze drying, all aimed at improving microbial viability and shelf stability. Moreover, the use of biostimulants and hydrogel-forming polymers is gaining attention as a sustainable replacement for conventional agricultural inputs [23].

### 3.3. Efficacy and Safety Profiles

While the efficacy of a microbial agent is a determining factor in its industrial development and practical application, it is equally critical to evaluate the potential effects that its active compounds may exert on humans and non-target organisms within the broader ecosystem. For instance, certain microbial pesticide strains used in agriculture may pose health risks, particularly to immunocompromised individuals who are more susceptible to opportunistic infections. Some members of the Bacillaceae family, notably Bacillus cereus and Bacillus anthracis, have been associated with such infections and can lead to serious health complications [37,38]. In contrast, several subspecies such as *B. thuringiensis* and *B. subtilis*, widely used in biocontrol and bioremediation, have not been linked to any reported infections in humans or animals to date. This contrast underscores the importance of comprehensive risk assessment before field deployment. Furthermore, antibiotic metabolites produced by *Pseudomonas fluorescens* have demonstrated toxicity toward certain earthworm species, as confirmed through controlled laboratory bioassays evaluating their impact on non-target soil fauna. Understanding these complex ecological interactions is therefore essential to ensure the safe and responsible application of microbial agents in agricultural and industrial settings [1]. Similarly, plant-derived products such as *Yucca schidigera* extract, which is rich in saponins, exemplify how a single compound can serve dual roles—promoting seed germination while simultaneously acting as a natural fungicide [39].

## 4. Synergistic Approaches in Biocontrol Strategies

Synergistic approaches that combine microbial biocontrol agents with phytochemical biopesticides represent promising strategies for achieving effective and durable crop protection [8]. Biological control broadly refers to the use of living organisms or their metabolic by-products to mitigate the effects of plant pathogens. Microorganisms serving as biological control agents (BCAs) act through diverse mechanisms, including parasitism, antibiosis, and competition, with their efficacy being strongly influenced by environmental and physiological conditions. Phytochemical biopesticides, derived from plant secondary metabolites such as essential oils, alkaloids, flavonoids, and coumarins extracted from herbs, spices, or aromatic plants [14], exhibit potent insecticidal, fungicidal, nematicidal, and bactericidal activities. These natural products offer greater safety to crops, consumers, and the environment than conventional chemical pesticides. When microbial antagonists are combined with plant-derived biochemicals, synergistic or additive effects have been consistently observed against a wide range of phytopathogens and insect pests across diverse crop systems [4,5]. Broader implementation of such synergistic biocontrol strategies could substantially reduce dependence on synthetic agrochemicals and promote the transition toward sustainable plant protection [40].

### 4.1. Combining Microbial and Phytochemical Agents

**Figure 3:**
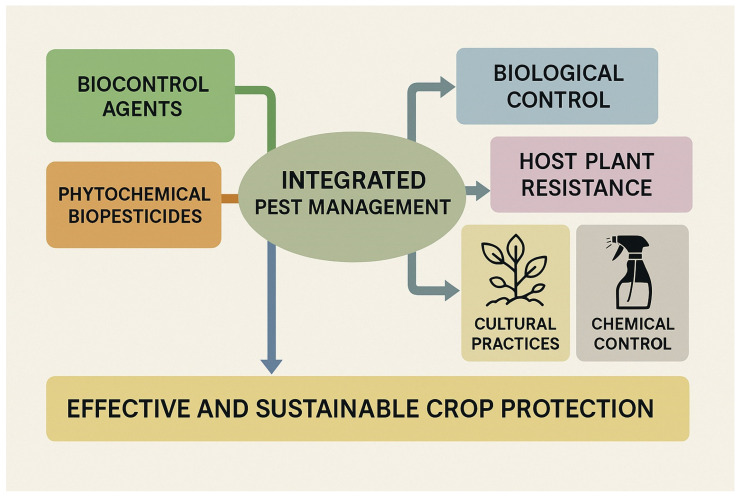
Integrated Pest Management (IPM) framework illustrating the combination of eco-friendly approaches, including biocontrol agents, phytochemical biopesticides, biological control, host plant resistance, cultural practices, and reduced chemical control, all contributing to effective and sustainable crop protection.

Integrated Pest Management (IPM) has gained unprecedented momentum and is now widely recognized as a universal and holistic framework for the sustainable control of plant diseases. IPM strategies integrate multiple environmentally responsible crop protection approaches, including biological control, host plant resistance, physical and cultural practices, and the judicious use of chemical agents, all aligned with the principles of sustainability, food safety, and environmental protection (Figure 3). A fundamental prerequisite for the success of IPM is that ecofriendly alternatives must demonstrate equal or superior efficacy compared to synthetic pesticides to ensure practical adoption by farmers. Recent studies have shown that biocontrol agent (BCA)-based strategies, when properly managed, can match or even surpass the performance of conventional chemical controls, thereby advancing the development of efficient and sustainable crop protection systems [9,11].

Biocontrol agents (BCAs) are microorganisms employed to suppress or eliminate phytopathogens, while phytochemical biopesticides are plant-derived compounds evaluated for their effectiveness in crop protection [14]. Studies investigating the combined application of these two strategies have consistently demonstrated synergistic interactions that enhance the suppression of pathogen development and disease progression, resulting in greater efficacy than when either agent is applied individually [41]. At the mechanistic level, several complementary processes help explain these synergistic outcomes. Many phenolic acids, flavonoids, and terpenoids released from plant tissues at sublethal concentrations act as chemoattractants and signaling molecules for beneficial bacteria and fungi. They can enhance chemotaxis, root-surface attachment, and biofilm formation in genera such as *Pseudomonas*, *Bacillus*, and *Trichoderma*, thereby improving rhizosphere colonization and spatial proximity to the pathogen. In addition, specific phytochemicals modulate microbial gene expression, activating biosynthetic gene clusters involved in the production of antibiotics, lipopeptides, volatile organic compounds, and cell-wall-degrading enzymes, which strengthens direct antagonism. Several phytochemicals also prime plant defense signaling through salicylic-acid, jasmonic-acid, and ethylene pathways; when combined with microbially induced systemic resistance, this results in faster and stronger activation of defense-related genes and metabolites. Surfactant-like compounds such as saponins can further increase pathogen membrane permeability and thus sensitize them to microbial toxins. Together, these effects create multi-layered interference with pathogen infection cycles and help explain why microbial–phytochemical combinations frequently outperform single-agent applications [4,5,40,41,42,43,44]. While synergistic formulations offer promising results, their combined use also introduces specific ecological and formulation-related challenges that require careful management. In soil ecosystems, both microbial and phytochemical components interact dynamically with indigenous microbiota, and unintended antagonism can occasionally occur. For example, high concentrations of phenolic or terpenoid compounds may inhibit spore germination or enzymatic activity of beneficial strains such as *Bacillus subtilis* or *Trichoderma harzianum*. Similarly, some essential oils can disrupt soil microbial community structure by selectively suppressing sensitive taxa, altering nutrient cycling and root–microbe signaling. To mitigate these risks, compatibility and dosage standardization are crucial. Laboratory screening and microcosm assays help identify threshold concentrations that enhance biocontrol efficacy without impairing microbial viability or soil functions. The development of co-formulated encapsulation systems or sequential application protocols (e.g., staggered microbial and phytochemical delivery) can prevent direct antagonistic contact and ensure stability under field conditions. Furthermore, tank-mix compatibility testing and clear labeling standards—similar to those used for chemical–biological mixtures—are essential for regulatory approval and practical deployment. Ultimately, the success of combined biocontrol approaches depends on rigorous formulation design, risk assessment, and field validation, ensuring that synergistic benefits outweigh potential environmental trade-offs [8,9,28,39,42]. The effectiveness and reliability of combined biocontrol strategies often increase with the diversity in the modes of action or environmental persistence between the two components. Many microorganisms exert multiple biocontrol mechanisms, including parasitism, competition, production of bioactive metabolites, and induction of plant resistance, which together provide robust and sustainable pathogen suppression. Numerous studies have explored these diverse mechanisms and materials to evaluate their potential as innovative, eco-friendly solutions for disease management. Despite the remarkable progress achieved with chemical fungicides since their widespread adoption after the 1940s, their extensive use raises major concerns regarding food safety, environmental sustainability, and human health. Many synthetic fungicides exhibit toxic, mutagenic, carcinogenic, or teratogenic effects and contribute to ozone depletion. Furthermore, the accumulation of chemical residues in food products can trigger chronic or acute physiological disorders in consumers. Therefore, there is an urgent need for effective, sustainable, and environmentally benign alternatives that safeguard both human health and ecological integrity. At this stage, biopesticides—including living microorganisms, plant extracts, essential oils, and their bioactive fractions—have emerged as promising substitutes. These naturally derived materials emulate the biochemical defense mechanisms of plants and other organisms, providing innovative, safe, and short-lived solutions for modern crop protection.

### 4.2. Case Studies of Successful Synergies

Integrating complementary biological control approaches is essential for developing effective and economically viable strategies in sustainable crop protection. Complex, integrated disease management systems that provide broad-spectrum control and protect crops from seed to harvest represent the most promising direction for the future of pest management. The successful and long-term use of biocontrol methods depends on practices grounded in ecological understanding rather than simple chemical replacement. Combined applications of bacterial and fungal biocontrol agents—or formulations incorporating multiple Bacillus species that produce *Bacillus thuringiensis* toxins and lipopeptides—have demonstrated additive or synergistic effects against a range of insect pests and fungal pathogens. For example, enhanced antifungal synergy has been documented in combinations of arbuscular mycorrhizal fungi, *Bacillus pumilus*, and *Pseudomonas alcaligenes* [15]. However, biological control using single microorganisms often encounters limitations such as competition with native microflora, environmental fluctuations, and inconsistent efficacy under field conditions. To overcome these constraints, microbial consortia comprising multiple species with complementary mechanisms of action have emerged as one of the most promising strategies for improving both the consistency and magnitude of disease suppression [14]. Despite significant progress in research, commercialization of microbial consortia-based formulations remains limited, underscoring the need for further development and field validation to advance sustainable plant cultivation systems.

## 5. Challenges in Implementation

**Figure 4:**
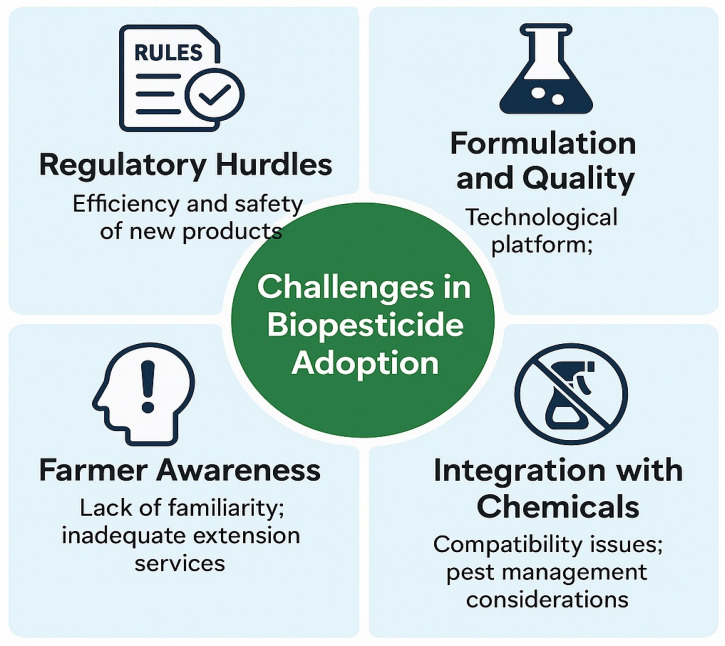
Key challenges limiting the adoption and commercialization of biopesticides. These include regulatory hurdles (efficiency and safety of new products), formulation and quality issues (technological platform and limited availability), farmer awareness (lack of familiarity and inadequate extension services), and integration with chemicals (compatibility issues and pest management considerations).

The development and large-scale application of biocontrol agents still face several critical scientific, regulatory, and practical challenges that restrict consistent field performance and wider commercialization. First, biological variability and environmental sensitivity—including temperature, humidity, soil pH, and UV exposure—often reduce efficacy under open-field situations [1,27]. Second, the mass production, stabilization, and formulation of living organisms or plant-derived compounds remain technically complex, requiring improved carrier materials, encapsulation technologies, and shelf-life optimization [22,23]. Third, non-harmonized regulatory frameworks among countries elevate the time and cost of product registration, hindering market entry [9,29]. Fourth, limited farmer awareness, inconsistent extension services, and lack of technical training restrict adoption, particularly in low-income regions [28]. These barriers can be mitigated through multi-tiered strategies: (i) advancing formulation technologies such as polymer-based encapsulation, nano-carriers, and co-culture consortia to improve stability and field persistence; (ii) developing region-specific microbial strains and phytochemical blends adapted to local agro-climatic conditions; (iii) establishing unified international regulatory guidelines and public–private partnerships to reduce approval costs and promote knowledge transfer; and (iv) strengthening farmer-training and outreach programs that demonstrate the economic and environmental benefits of biocontrol systems. A practical illustration of these challenges can be seen in the commercialization of *Trichoderma harzianum* and *Bacillus subtilis*-based products, among the most widely studied and marketed microbial agents worldwide. Despite proven laboratory efficacy against soil-borne pathogens such as *Rhizoctonia solani* and *Fusarium oxysporum*, field performance often fluctuates because of inconsistent spore viability, temperature and moisture sensitivity, and incompatibility with certain agrochemicals. *Trichoderma* formulations require precise moisture and carrier control during storage to maintain shelf life beyond six months, whereas *Bacillus* formulations may lose efficiency under prolonged exposure to high temperatures during transport or field application. From a regulatory perspective, the registration process for these microbial products differs substantially among regions. The European Union classifies microbial biocontrols under Regulation (EU) 2019/1009, demanding exhaustive toxicological and ecological risk assessments that are both time- and cost-intensive. In contrast, countries with less-defined frameworks may experience quality-control issues and inconsistent labeling. These practical examples demonstrate that both scientific optimization and policy harmonization are critical to achieving large-scale, reliable adoption of microbial biocontrol agents [45,46]. Biopesticides comprising microbials or phytochemicals are often regulated differently from synthetic counterparts. Microbial biopesticides face considerable regulatory hurdles because they are living organisms—one of the main bottlenecks for commercial exploitation and widespread adoption [1,22]. General regulatory challenges affecting biopesticide market penetration and growth relate to establishing the efficiency and safety of new microbial- or plant-based products (Figure 4). Studies have documented multiple unresolved issues restricting the wider adoption of microbial biocontrol agents [2,9]. These challenges also mirror the situation for phytochemical biopesticides, since both depend on natural organisms (fungi or plants) that produce complex bioactive compounds. Hence, the development of phytochemical products—and their integration with microbial agents—requires a deeper understanding of factors influencing commercial scalability. Despite substantial R&D progress, market penetration for biofungicides remains comparatively low. Addressing this gap requires a robust technological platform to ensure consistent product quality, shelf stability, and reliable supply [28]. The availability of microbial control products is still limited, and many suffer from short shelf life and variable performance, discouraging their wider utilization. Insufficient farmer awareness and inadequate extension services contribute to improper dosages and application timing, sometimes leading to resistance development under intensive use. Integration of biocontrol agents with low doses of synthetic fungicides in IPM systems can mitigate these issues: such combinations protect seeds and seedlings, enhance overall fungicide effectiveness, and reduce dependency on highly toxic compounds. Incorporating diverse biocontrol agents alongside compatible synthetic fungicides thus provides flexible and safer alternatives for managing a wide spectrum of phytopathogens. Nevertheless, operational challenges persist. Optimum storage conditions and application timing differ from those of chemical pesticides; many biocontrol agents must be applied earlier and are less tolerant of adverse weather. If management of one pest requires a chemical incompatible with a biocontrol organism, integrated use becomes impractical, requiring alternative strategies. Compatibility studies have often been limited or inconsistent [47]. Consequently, information gaps continue to complicate decision-making. At the same time, evolving regulatory frameworks, crop-management practices, and consumer expectations increasingly favor the integration of biocontrol. Label restrictions on chemical pesticides, the emergence of new invasive pests, and growing consumer preference for residue-free produce improve the relative suitability and market opportunity for microbial and phytochemical products. Moreover, regulatory emphasis on pollinator protection opens new possibilities for biocontrol strategies that are pollinator-safe and compatible with biodiversity-enhancing practices such as perennial habitat strips. The expanding diversity of biocontrol products—especially bacterial and fungal inoculants—combined with growing evidence of positive field performance, suggests that biocontrol will continue to evolve into a desirable, cost-effective, and ecologically sound pillar of modern pest-management systems.

### 5.1. Regulatory Hurdles

Introducing biopesticides to the market, even well-established ones, remains costly, time-consuming, and procedurally complex. Regulatory scrutiny and farmer conservatism often create higher entry barriers for biopesticides than for conventional pesticides. Although extensive data demonstrate that microbial biopesticides are generally safe, the regulation of phytochemical biopesticides remains inconsistent across jurisdictions [1]. The lack of a uniform global framework impedes product access and limits market penetration, even amid rising demand [9]. Regulatory agencies—including the European Union and the U.S. Environmental Protection Agency—are engaged in ongoing efforts to refine policies, streamline data requirements, and harmonize risk-assessment methodologies. Establishing a coherent, transparent, and science-based regulatory structure is now recognized as a cornerstone for sector growth and long-term sustainability [29]. Continued collaboration among governments, industry, and academia is essential to ensure that safety standards are maintained without stifling innovation.

### 5.2. Market Acceptance and Economic Viability

Beyond technical and regulatory barriers, economic feasibility and farmer perception remain decisive for adoption. For biocontrol to achieve widespread use, microbial and phytochemical agents must be cost-competitive and demonstrably effective. Although their field efficacy can equal that of synthetic pesticides, production, formulation, and distribution expenses often make them more expensive initially [1,22]. In highly competitive farming systems, short-term profitability outweighs long-term ecological benefits. Consequently, biocontrol products occupy niche segments such as organic or high-value crops, representing a relatively small proportion of global acreage. Despite this limitation, increased consumer concern over food safety and environmental protection has created new market incentives for residue-free produce. Expanding economic incentives, government subsidies, and eco-labeling programs can improve cost–benefit ratios for farmers. The promotion of green-credit schemes, carbon-neutral branding, and demonstration farms can further enhance visibility and market share. Integrating microbial and phytochemical agents within integrated pest-management (IPM) frameworks ensures efficiency while lowering chemical dependency, aligning economic gains with sustainability targets.

## 6. Field Trials and Practical Applications

The successful implementation of sustainable crop protection programs fundamentally depends on the design, execution, and analysis of well-structured field trials. At present, there are no standardized experimental methodologies specifically developed to evaluate the synergistic effects of combined microbial biocontrol agents and phytochemical biopesticides under field conditions. Moreover, limited datasets are available to guide the large-scale development and validation of such integrated systems. Nonetheless, the aggregation of unpublished data from small, independent field experiments has provided preliminary evidence supporting the potential benefits of integrating microbial and phytochemical agents [42]. This emerging trend is consistent with recent findings showing that combinations of plant growth-promoting bacteria and biostimulants can exert synergistic effects against abiotic stress, suggesting a similar potential in biotic stress mitigation [43,44]. However, extensive research is still required before such synergistic approaches can be standardized and widely adopted in agricultural practice. Large-scale field trials remain essential to validate the efficacy, reproducibility, and scalability of novel synergistic formulations. Over the past decade, numerous studies have demonstrated that the efficacy of microbial biocontrol agents can be substantially enhanced when co-applied with piperitenone oxide, a phytochemical compound exhibiting both repellent and insecticidal activity [1]. Such combined applications offer a cost-effective and intensified biocontrol strategy, aligning with the global demand for sustainable, eco-efficient crop protection solutions. These field observations are consistent with the mechanistic model outlined in Section 4, where phytochemicals enhance microbial chemotaxis, colonization, antibiotic production, and plant defense priming, resulting in multi-layered suppression of target pests [42,43,44]. In addition to efficacy, key parameters for optimization include impact on non-target organisms, environmental persistence, and spectrum of control.

### 6.1. Designing Effective Field Trials

**Figure 5:**
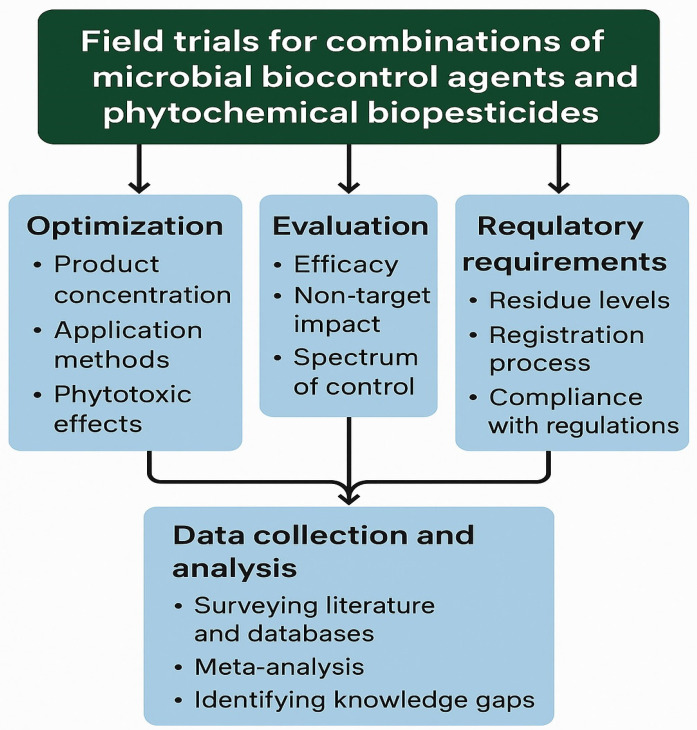
Workflow for field trials combining microbial biocontrol agents and phytochemical biopesticides. Essential steps include optimization (concentration, application methods, phytotoxicity), evaluation (efficacy, non-target impact, spectrum of control), regulatory requirements (residue levels, registration process, compliance), and data collection and analysis (literature surveys, meta-analysis, identification of knowledge gaps).

Field trials are essential for validating the laboratory or greenhouse efficacy of novel plant disease control products under real-world conditions (Figure 5). They enable the optimization of product concentration, application methods—including timing, frequency, and delivery mode—and facilitate the detection of any potential phytotoxic effects. The outcomes of these trials form the scientific foundation for product registration dossiers. Commercial formulations based on plant extracts typically adhere to maximum residue levels (MRLs) established for major active ingredients that naturally occur in food and beverages. However, hydrodistilled essential oils may exhibit phytotoxic effects, even at diluted concentrations; therefore, post-application testing, extending to the time of first harvest, is strongly recommended prior to commercialization. In contrast, extracts of unprocessed botanicals are generally safe with respect to food residues, provided that good agricultural and manufacturing practices are observed. Exposure of pollinators, such as bees, to microbial or plant-extract-based biopesticides is generally non-hazardous, as microbial formulations typically exhibit short persistence and high repellency [1]. Globally, the regulatory approval of new biopesticidal products is overseen by national and international authorities, requiring submission of comprehensive registration dossiers in standardized formats. These dossiers are evaluated under transparency and confidentiality frameworks, and final approval depends on rigorous assessments of efficacy, toxicity, persistence, and environmental safety. While multi-ingredient formulations may be subdivided into component dossiers [9], the registration timeline is often longer for new semiochemicals compared to microbial or botanical biopesticides. Within the European Union, compliance with EU Regulation 2021/383/EU on biocides is mandatory. Extracted compounds must conform to Annex II or III to qualify for “natural origin” status, and regulatory eligibility must be verified through the relevant national authority before submission. The European Food Safety Authority (EFSA) has already excluded most toxic compounds listed under Annex II, while those posing high environmental or health risks are restricted under Annex III. Current EU priorities emphasize the production technologies and purity standards of naturally derived compounds to ensure compliance with plant protection legislation. Recent studies highlight the promise of combined microbial–phytochemical biopesticide formulations. For example, the fungal entomopathogen *Metarhizium anisopliae* and the botanical insecticide pyrethrum have shown synergistic activity against the European bean aphid (Aphis fabae), markedly increasing insect mortality rates [6,7]. Similarly, in *Botrytis cinerea*, the addition of botanical formulations to microbial biofungicides has been shown to reduce fungicide resistance development. Moving forward, the integrated use of microbial biocontrol agents and phytochemical biopesticides is expected to become a core component of sustainable pest management in both arable and orchard cropping systems.

### 6.2. Data Collection and Analysis

Data collection and synthesis can be effectively achieved through systematic surveys of existing literature and specialized databases on microbial biocontrol agents and phytochemical biopesticides. Applying comprehensive analytical approaches, such as rigorous meta-analyses, enables researchers to critically evaluate the efficacy, mechanisms of action, and compatibility of diverse biocontrol strategies [48,49]. By extracting and integrating key insights from reliable scientific sources and peer-reviewed studies, researchers can identify significant knowledge gaps and pinpoint specific areas where experimental findings may be translated into practical field applications. This integrative, evidence-based approach ensures a holistic understanding of current advances and challenges in sustainable biocontrol, thereby facilitating the development of optimized and scalable crop-protection frameworks.

## 7. Future Directions in Sustainable Crop Protection

Modern agriculture faces escalating challenges, including rising food demand, limited arable land, and increasing pest and pathogen pressure. The development of resistance among phytopathogens has reduced the effectiveness of synthetic agrochemicals, creating an urgent need for eco-friendly alternatives. Hence, sustainable development of novel bio-based pesticides that protect yield and environmental quality has become essential. Microbial biocontrol agents (MBCAs) bridge effective pest control with sustainability but still face obstacles in manufacturing, formulation, and field performance that limit large-scale commercialization [5]. At the same time, phytochemical biopesticides represent valuable synergistic partners because of their potent antimicrobial activity and reduced ecological impact. Formulating synergistic MBCA–phytochemical combinations therefore offers a promising and innovative strategy for resilient, sustainable crop protection [8,9,50].

### 7.1. Innovations in Biocontrol Technologies

Phytochemical biopesticides—plant-derived compounds from essential oils, leaves, stems, or seeds—serve as effective, lower-toxicity alternatives to synthetic pesticides. However, market acceptance, regulatory stringency, and production costs still hinder broader utilization. When microbial biocontrol agents and phytochemical biopesticides are combined, their complementary actions can enhance efficacy while reducing environmental load. Encouraging regulatory flexibility and promoting integrated biocontrol approaches could therefore strengthen adoption and advance sustainable agriculture [9,50].

### 7.2. Integrating Biocontrol in Agricultural Practices

Microbial consortia containing complementary species or strains with different antagonistic mechanisms represent the next step in BCA development. Although laboratory results demonstrate improved pathogen suppression, few multi-strain formulations are yet commercialized. Achieving long-lasting, reliable formulations remains a key challenge. Continued optimization of strain compatibility, carrier materials, and field delivery methods will be vital to unlock the full potential of consortium-based biocontrol and ensure a sustainable future for plant protection.

## 8. Environmental Impact of Biocontrol Agents

Microbial biocontrol agents are recognized as environmentally sustainable components of modern pest-management programs, directly aligning with the goals of eco-friendly agriculture [51]. Commercially available products derived from beneficial microbes are formulated to minimize ecological risks while maintaining effective crop protection. Likewise, phytochemical biopesticides—notably essential-oil-based compounds and plant secondary metabolites—are valued for their short environmental persistence, biodegradability, and low toxicity compared with conventional agrochemicals [8,9,52]. Their use enables farmers to safeguard crops while simultaneously promoting ecosystem health, conserving beneficial organisms, and minimizing soil and water contamination. Taken together, these bio-based technologies form an essential environmental pillar of integrated pest-management (IPM) strategies, offering protection that harmonizes agricultural productivity with ecological integrity.

### 8.1. Ecosystem Health Considerations

Sustainable pest control requires that microbial and plant-derived biopesticides remain compatible with biodiversity and ecosystem balance. Compatibility ensures that the introduction of beneficial agents does not inadvertently disturb native microbial communities or higher-trophic organisms. Some microbial–phytochemical combinations exhibit clear synergism; for instance, *Fusarium oxysporum* biocontrol agents combined with *Cymbopogon citratus* (lemongrass) essential oil enhance pathogen suppression and plant resilience. In contrast, other pairings may prove antagonistic, such as *Aspergillus* with *Ocimum basilicum* (basil) or *Bacillus* with *Syzygium aromaticum* (clove) and *Cinnamomum zeylanicum* (cinnamon) essential oils [8]. This incompatibility stems from the strong antimicrobial constituents of these oils—particularly eugenol and cinnamaldehyde—which disrupt bacterial cell membranes, inhibit ATPase activity, and suppress spore germination in *Bacillus* spp. These effects compromise spore viability and metabolic activity, thereby reducing the biocontrol efficiency of *Bacillus*-based formulations. Experimental evidence supports this observation: similar inhibitory interactions between *Bacillus subtilis* and phenolic-rich essential oils have been documented both in vitro and in bioformulation trials [28,39,42]. Conversely, numerous beneficial interactions have also been reported. Plant-derived compounds can stimulate microbial colonization or metabolic activity, while bacteria such as *Pseudomonas* sp., *Bacillus subtilis*, and *Bacillus velezensis* improve the solubilization and uptake of phytochemical constituents [14]. Such positive feedback highlights how carefully optimized microbial–phytochemical formulations can reinforce ecological equilibrium rather than disrupt it. Overall, understanding the biochemical basis of compatibility and antagonism is crucial for designing next-generation formulations that maximize pest-control efficiency without compromising environmental integrity.

### 8.2. Biodiversity and Biocontrol

A wide range of microbial groups exhibit potent biocontrol activity in horticultural and field crops through mechanisms such as antibiosis, induced systemic resistance (ISR), competition, and mycoparasitism. These mechanisms confer broad-spectrum action, environmental safety, and relatively simple production procedures. By reducing dependence on synthetic pesticides, microbial and phytochemical biopesticides collectively support agro-ecosystem biodiversity and restore soil microbial balance. However, commercial expansion continues to face regulatory and economic bottlenecks, as discussed earlier, emphasizing the need for environmentally informed innovation in product development [8,9]. Combining microbial biocontrol with phytochemical formulations not only enhances field efficacy but also reduces the overall ecological footprint of crop-protection regimes. Phytochemicals—natural plant metabolites that protect against insects and pathogens—are generally extracted from plant tissues, yet their seasonal variability can affect consistency in composition and supply. Plant-tissue-culture technologies provide a sustainable, reproducible alternative, ensuring the continuous production of uniform bioactive compounds for large-scale biopesticide manufacturing [53]. This approach minimizes pressure on wild plant resources while maintaining genetic and chemical fidelity, reinforcing the environmental sustainability of phytochemical production. By integrating microbial and phytochemical strategies within well-regulated frameworks, agriculture can achieve a dual benefit: improved pest control and strengthened ecosystem resilience.

## 9. Economic Implications of Biopesticide Use

Economic considerations are central to the large-scale adoption of microbial and phytochemical biopesticides. Despite their agronomic and environmental advantages, the cost-effectiveness of biopesticides in crop protection remains difficult to quantify because of limited data on price per unit area, optimal dosage, and irrigation or application requirements. Yields are sometimes perceived as lower compared with chemical pesticides, and most farmers remain reluctant to adopt biopesticides primarily because of higher purchase prices [9,22]. Although biopesticides may initially cost more than synthetic counterparts, this premium can be justified when externalities are considered. Chemical pesticides impose hidden costs through disposal requirements, environmental degradation, and health risks, whereas biopesticides offer safer, biodegradable, and socially acceptable alternatives. Economic, social, and policy factors thus jointly shape market acceptance and determine adoption rates [29]. Government initiatives to raise awareness, provide subsidies, and promote eco-labeling play a critical role in accelerating adoption. Integration of biopesticides into integrated pest-management (IPM) programs can further offset costs, reduce resistance, and improve overall system profitability. Moreover, as consumers become more conscious of sustainability and food safety, demand for organic or residue-free produce is expected to increase willingness to pay. Ultimately, the success of microbial and phytochemical biopesticides will depend on their economic viability, the scale of governmental support, and farmers’ perception of long-term benefits relative to short-term costs.

### 9.1. Cost–Benefit Analysis

Cost–benefit analyses (CBAs) provide a structured method to evaluate the profitability and sustainability of pest-management options, quantifying the economic return over time [9]. A representative partial-budget analysis compared three scenarios: (i) expert-recommended integrated strategies, (ii) commercial grower practices, and (iii) assumed biopesticide adoption. The expert-recommended plan—incorporating rotation of pesticides with different modes of action—achieved effective pest suppression but incurred higher production costs, whereas conventional grower strategies achieved lower expenses but also reduced long-term efficacy [54]. Biopesticides such as Met 52 and QRD 452, both registered microbial formulations, have been examined through CBAs. Their slower mode of action and higher production costs initially result in elevated prices, yet these premiums typically decline as more competitors enter the market and economies of scale improve. In addition, CBAs frequently demonstrate that environmental safety and soil-health benefits provide non-monetary gains often overlooked in conventional analyses [22]. Recent studies highlight that microbial, phytochemical, and nano-biopesticides contribute to maintaining soil–microbiome balance, reducing carbon footprints, and supporting integrated crop-management frameworks [55]. Incorporating microbial inoculants not only enhances pest control but also improves nutrient availability and crop vigor, thereby increasing long-term economic resilience. A well-designed regulatory and developmental framework thus remains pivotal for unlocking the full market potential of these technologies. Furthermore, targeted biocontrol solutions can provide economically viable alternatives for specific pest problems—for example, biopesticides have proven effective against the pine processionary moth, demonstrating how environmentally benign options can substitute for costly chemical interventions.

### 9.2. Impact on Farming Practices

The adoption of microbial and phytochemical biopesticides is transforming traditional farming systems, shifting production toward more sustainable and diversified models. Farmers employing biopesticides report reduced reliance on synthetic chemicals and a corresponding decline in problems associated with residue contamination of produce and the environment. This transition allows broader crop rotation, diversification of varieties, and expansion of cultivated area while maintaining soil health and productivity. Enhanced biodiversity arising from diversified cropping systems promotes natural pest regulation and ecological stability. Microbial biocontrol agents (MBAs) provide multiple complementary mechanisms—including competition, antibiosis, induced resistance, and parasitism—that together strengthen crop resilience. Distinct microbial taxa contribute unique benefits: *Bacillus* spp. induce systemic resistance and synthesize antimicrobial metabolites; *Trichoderma* spp. parasitize pathogens and compete for niches; *Pseudomonas* spp. regulate plant defense genes and improve nitrogen cycling; *Rhizobium* spp. fix atmospheric nitrogen and attract beneficial organisms [8,51,56]. These ecological services translate into tangible economic and social advantages, including reduced pesticide expenditure, improved yield stability, and enhanced consumer trust in eco-labeled products. The cumulative effect is a positive feedback loop where environmental sustainability and economic profitability reinforce each other, paving the way for resilient agro-economic systems built on biopesticide integration.

## 10. Consumer Perspectives on Biopesticides

The growing emphasis on sustainable crop protection reflects rising consumer demand for environmentally responsible food production. As global populations increase, agricultural practices must meet food needs without compromising ecosystem health. Biocontrol and biopesticide technologies represent a viable, economically sound pathway toward producing safe, residue-free food, aligning with public concerns about human health, environmental impact, and farm-worker safety. Despite these advantages, public awareness of biopesticides remains limited, which restricts their mainstream acceptance. Understanding consumer perceptions, motivations, and concerns is vital for expanding their global market share. Educational programs, transparent labeling, and demonstration trials can help consumers and producers recognize the environmental and health benefits of microbial and phytochemical biopesticides, fostering trust in these sustainable technologies [29].

### 10.1. Public Awareness and Education

Many farmers, particularly smallholders and local producers, face barriers such as limited financial access, weak market infrastructure, and insufficient technical guidance. These factors hinder the adoption of sustainable pest-management practices [57]. Expanding affordable credit, training programs and extension services is critical for enabling farmers to transition from conventional agrochemicals to biopesticide-based systems. Public-awareness campaigns—supported by agricultural ministries, cooperatives, and NGOs—should emphasize safe pesticide handling, residue management, and integrated pest management (IPM) principles. For example, participatory initiatives in several Asian and Mediterranean countries have shown that farmer-field schools and consumer-education drives significantly reduce chemical pesticide misuse and improve acceptance of biocontrol products [9,58]. Developing consistent national and regional frameworks, complemented by public–private partnerships, can strengthen both consumer confidence and market transparency. These proactive actions ensure that sustainable production benefits farmers, retailers, and consumers alike, contributing to an environmentally conscious agri-food chain.

### 10.2. Consumer Acceptance Studies

Consumer attitudes toward biopesticide use are increasingly shaping agricultural policy and marketing strategies. Studies indicate that when consumers receive clear, science-based information, acceptance and willingness to pay for biopesticide-treated produce increase substantially. Effective communication through certification labels, awareness campaigns, and retail interventions enhances consumer trust and influences purchase intentions for organic or residue-free products [59,60]. However, challenges persist. Biopesticides may show slower action or inconsistent performance compared with synthetic pesticides, occasionally leading to skepticism about efficacy. In addition, certain phytochemical-based biopesticides, particularly those derived from essential oils rich in phenolics and terpenoids such as eugenol, thymol, or cinnamaldehyde, can exhibit dose-dependent toxicity toward beneficial microbes or non-target organisms when used at excessive concentrations or without proper formulation control. This phenomenon does not contradict their overall safety profile but highlights the need for careful standardization, optimized dosages, and good-practice application guidelines to maintain selectivity and environmental compatibility. Ongoing comparative studies are therefore essential to evaluate how information framing, demonstration projects, and pricing strategies affect consumer acceptance across regions. Integrating microbial biological-control agents with phytochemical biopesticides remains a promising path forward, addressing consumer concerns about safety while improving pest-control efficiency and supporting global goals for sustainable, health-oriented agriculture [1,9].

## 11. Conclusions

Sustainable crop protection encompasses a comprehensive set of innovative technological approaches aimed at minimizing dependence on synthetic agrochemicals while enhancing plant resilience against a wide range of biotic stress factors. These stressors—originating from pests, pathogens, and diseases—continue to threaten global food security by reducing yield, quality, and environmental stability. Within this evolving agricultural landscape, two major categories of biopesticides have emerged as central to sustainable farming: microbial biocontrol agents (MBCAs) and phytochemical biopesticides. Both are derived from naturally occurring chemical signals that mediate inter-organismal communication, forming the foundation of eco-friendly crop protection systems. The integration of these two biological strategies offers a synergistic platform that combines the precision of microbial antagonism with the chemical diversity of plant metabolites. Together, they can strengthen crop defense mechanisms, reduce the environmental footprint of agriculture, and support circular bioeconomy goals. The key insights of this review emphasize that synergistic microbial–phytochemical applications can: (i) enhance pest and disease suppression through complementary molecular and physiological mechanisms; (ii) mitigate resistance development and improve soil biodiversity; (iii) reduce dependence on hazardous synthetic inputs; and (iv) foster regulatory, ecological, and economic sustainability within integrated pest management systems. To realize this potential, future research should prioritize: mechanistic exploration of microbial–phytochemical interactions at genomic, transcriptomic, and metabolomic levels to unravel pathways that enhance colonization, signaling, and pathogen inhibition; innovative formulation and delivery systems, such as nano-encapsulation and biofilm-assisted carriers, to ensure field stability, compatibility, and controlled release of active components; long-term ecological monitoring to evaluate non-target effects, soil health, and microbiome dynamics under combined applications; modeling and digital decision-support tools integrating remote sensing and AI technologies to optimize dosage, timing, and environmental safety. At the policy and regulatory level, harmonized frameworks are urgently needed to support registration of multi-component biological products, accompanied by global guidelines on efficacy testing, labeling, and safety assessment. Policymakers should incentivize the transition toward biological inputs through research funding, farmer training programs, and tax or subsidy schemes that encourage adoption. Strengthened collaboration between academia, industry, and international agencies will accelerate the scaling of these eco-compatible innovations. In summary, the synergistic integration of microbial biocontrol agents and phytochemical biopesticides represents one of the most promising frontiers in sustainable agriculture. By coupling mechanistic understanding with policy reform and technological innovation, future research and practice can deliver resilient, profitable, and environmentally balanced crop production systems, ensuring food security in the face of global change.

## Data Availability

No new data were created or analyzed in this study. Data sharing is not applicable to this article.
